# A novel nomogram and recursive partitioning analysis for predicting cancer-specific survival of patients with subcutaneous leiomyosarcoma

**DOI:** 10.1038/s41598-024-53288-6

**Published:** 2024-02-04

**Authors:** Qiang Ji, Hua Hu, Shulian Li, Jun Tang

**Affiliations:** 1 Department of Aesthetic Plastic Surgery, West China School of Public Health and West China Fourth Hospital, Sichuan University, Chengdu, 610041 China; 2 Department of Burn and Plastic Surgery, West China Hospital, Sichuan University, Guoxue Alley, Wuhou District, Chengdu, 610041 China; 3 Department of Thyroid Surgery, West China Hospital, Sichuan University, Guoxue Alley, Wuhou District, Chengdu, 610041 China

**Keywords:** Diseases, Risk factors

## Abstract

Accurately predicting prognosis subcutaneous leiomyosarcoma (LMS) is crucial for guiding treatment decisions in patients. The objective of this study was to develop prediction models for cancer-specific survival (CSS) in patients with subcutaneous LMS. The collected cases of diagnosed subcutaneous LMS were randomly divided into a training cohort and a validation cohort at a 6:4 ratio based on tumor location and histological code. The X-tile program was utilized to determine the optimal cutoff points for age index. Univariate and Cox multivariate regression analyses were conducted to identify independent risk factors for subcutaneous LMS patients. Nomograms were constructed to predict CSS, and their performance was assessed using C-index and calibration plots. Additionally, a decision tree model was established using recursive partitioning analysis to determine the total score for CSS prediction in subcutaneous LMS patients based on the nomogram model. A total of 1793 patients with subcutaneous LMS were found. X-tile software divides all patients into ≤ 61 years old, 61–82 years old, and ≥ 82 years old. The most important anatomical sites were the limbs (including the upper and lower limbs, 48.0%). Only 6.2% of patients received chemotherapy, while 44% had a history of radiotherapy and 92.9% underwent surgery. The independent risk factors for patients with subcutaneous LMS were age, summary stage, grade, and surgery. CSS was significantly decreased in patients with distant metastases, which showed the highest independent risk predictor (HR 4.325, 95% CI 3.010–6.214,* p* < 0.001). The nomogram prediction model of LMS was constructed based on four risk factors. The C-index for this model was 0.802 [95% CI 0.781–0.823] and 0.798 [95% CI 0.768–0.829]. The training cohort's 3-, 5-, and 10-year AUCs for CSS in patients with subcutaneous LMS were 0.833, 0.830, and 0.859, and the validation cohort's AUC for predicting CSS rate were 0.849, 0.830, and 0.803, respectively. Recursive segmentation analysis divided patients into 4 risk subgroups according to the total score in the nomogram, including low-risk group < 145, intermediate-low-risk group ≥ 145 < 176, intermediate-high-risk group ≥ 176 < 196, and high-risk group ≥ 196; The probability of the four risk subgroups is 9.1%, 34%, 49%, and 79% respectively. In this retrospective study, a novel nomogram or corresponding risk classification system for patients with subcutaneous LMS were developed, which may assist clinicians in identifying high-risk patients and in guiding the clinical decision.

## Introduction

Skin leiomyosarcoma (LMS) is a rare type of cutaneous mesenchymal sarcoma, accounting for only 11.4% of all soft tissue sarcomas^1,2^. It can be further categorized into two primary subtypes: primary dermal LMS and subcutaneous LMS. Dermal LMS originates from the vertical hair muscles in the dermis, while subcutaneous LMS may arise from smooth muscles associated with arteries and veins^3–6^. Tumor depth has been identified as a significant adverse prognostic indicator, with subcutaneous LMS exhibiting lower overall 5-year survival rates and shorter distant disease-free survival periods^7^. Although distant metastasis is uncommon after complete resection of cutaneous LMS, there have been reports of local recurrence and metastasis^8,9^. Compared to more common non-melanoma skin cancers, the recurrence rate of skin LMS remains high, ranging from 0 to 67% for primary dermal LMS and 19–40% for subcutaneous LMS^10^. Furthermore, subcutaneous LMS demonstrates a higher frequency of metastasis compared to dermal leiomyosarcoma.

Several independent predictors of survival have been identified in patients with LMS. For instance, high tumor grade, and tumor size ≥ 5 cm have been associated with decreased cancer-specific survival (CSS) in patients with extremity LMS^11^. However, another analysis found that only histologic grade independently influenced CSS^12^. Surgical resection is generally considered the primary treatment approach, while radiation therapy is often utilized in advanced stages, potentially impacting wound recovery for patients^13,14^. Despite studies suggesting that subcutaneous LMS should be managed with more extensive resection, adjuvant radiotherapy, and chemotherapy, there is still a lack of consensus guidelines for the clinical prediction and decision-making of such tumors.

This study aimed to construct and validate a prognostic nomogram for subcutaneous LMS by incorporating various factors. The developed nomogram provides a quantitative tool for estimating survival outcomes in patients. To further enhance clinical applicability, a decision tree model was constructed using recursive partitioning analysis (RPA). Combining the predictive power of the nomogram and the simplicity of the decision tree model, this study contributes to the improvement of personalized care for individuals diagnosed with subcutaneous LMS. The provided tools facilitate risk assessment and enable informed discussions between healthcare providers and patients regarding treatment strategies and prognostic expectations.

## Materials and methods

### Study population

Patient data were obtained from SEER (Surveillance, Epidemiology, and End Results) data. We identified cases of subcutaneous LMS diagnosed between the years 2000 and 2019.The inclusion criteria were based on the revised 8th edition of the American Joint Committee on Cancer (AJCC) guidelines. Specifically, the International Classification of Diseases for Oncology (ICD-O-3) histological type code used was 8890, corresponding to leiomyosarcoma. The site codes for extraction were as follows: C49.0-Conn for subcutaneous tumors in the head, face, and neck; C49.1-Conn for subcutaneous tumors in the upper limb and shoulder; C49.2-Conn for subcutaneous tumors in the lower limb and hip; and C49.6-Conn for subcutaneous tumors in the trunk, not otherwise specified. In addition, the behavior code used was "malignant". The exclusion criteria were as follows: population and treatment information were unknown or missing.

### Statistical analyses

**Figure 1:**
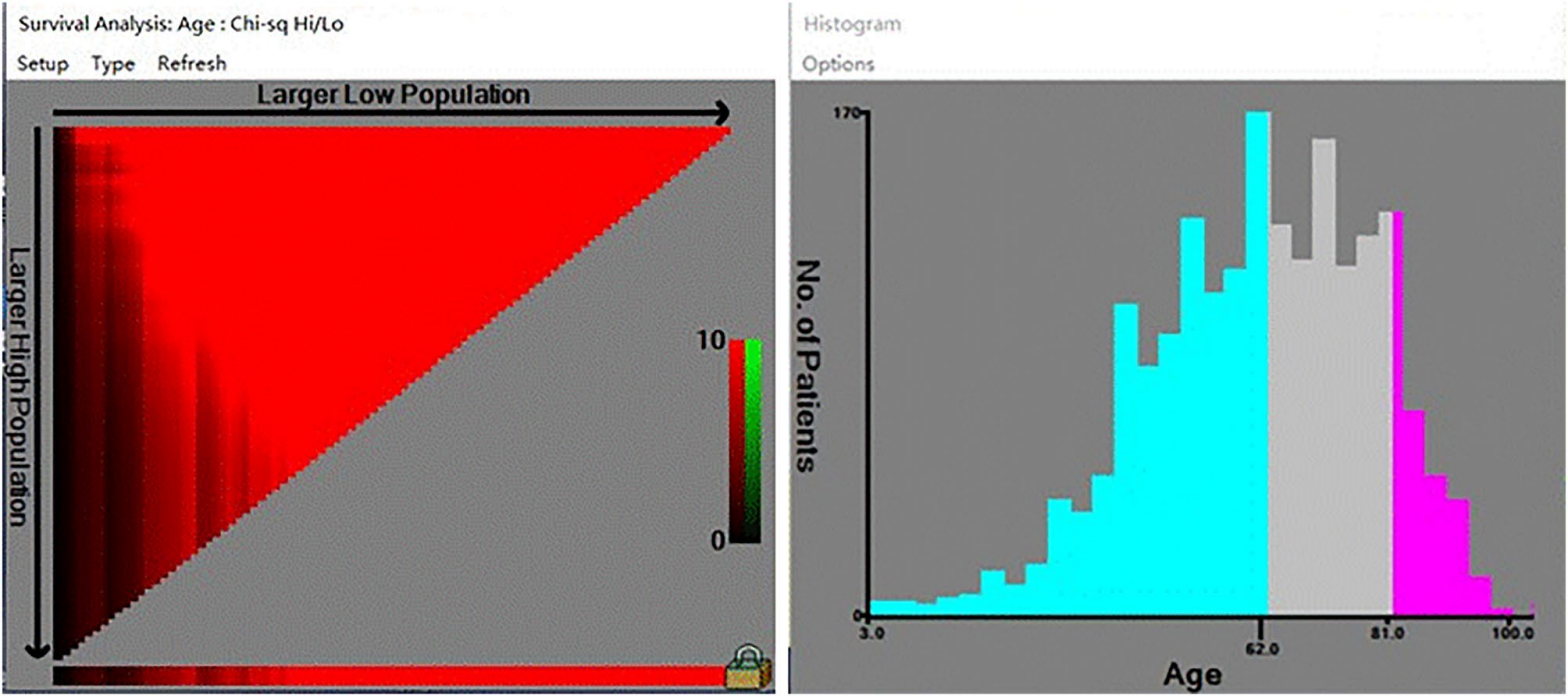
The optimal cut-off values for age (A-B) based on CSS were < 62 years, ≥ 62, < 81 years, and ≥ 81 years old.

X-tile software (Yale University, USA, Version 3.6.1) was employed to determine the optimal cut-off value for continuous variables. The selection of the best cut-off value was based on the highest chi-square value calculated using Kaplan–Meier survival analysis and logarithmic rank test. Utilizing the optimal threshold of age, we categorized all patients into three subgroups: ≤ 61 years old, 61–82 years old, and ≥ 82 years old (Fig. 1). Subsequently, we randomly divided the patients into a training cohort and a validation cohort, with a partition ratio of 6:4. The Kaplan–Meier analysis regression model was then employed to present the survival probability at various time intervals. To determine the factors influencing CSS in NM patients, Cox regression analysis was carried out, with SEER cause-specific death classification (Dead (attributable to this cancer dx)) serving as the dependent variable. All statistical analyses were performed using R 4.1.3 statistical software (http://www.rproject.org).

### Development and evaluation of a nomogram prediction model

The independent risk indicators identified were then incorporated into a nomogram to predict the survival probability at 3, 5, and 10-year CSS rates. The accuracy and discriminatory ability of the newly developed nomogram were evaluated using the AUC. The nomogram model was employed to calculate a total score for predicting the outcome of patients with subcutaneous LMS. Additionally, to facilitate risk assessment, a decision tree model was established by utilized the RPA method.

### Ethics

We acknowledge the SEER database for providing their platforms and contributors for uploading their meaningful datasets. SEER belongs to public databases. The patients involved in the database obtained ethical approval. Users can download relevant data for free for research and publish relevant articles. Our study is based on open data, so there are no ethical issues or other conflicts of interest.

## Results

### Study cohorts and patient characteristics

The study population comprised 1793 patients diagnosed with subcutaneous leiomyosarcoma (LMS) between 2000 and 2019, who met the inclusion criteria. The demographic and clinicopathological characteristics of these patients are presented in Table 1. Among the included patients, there were 837 individuals aged < 62 years, 707 patients aged between 62 and 81 years, and 249 patients aged > 82 years. Most patients were male, accounting for 56.9% of the population.

**Table 1:** Demographic and clinical characteristics of patients with subcutaneous soft tissue LMS for the training and validation cohorts.

	Overall (N = 1793)	Training cohort (N = 1075)	Valid cohort (N = 718)	*p-*value
Age				0.184
< 62 years	837 (46.7%)	502 (46.7%)	335 (46.7%)	
≤ 62, > 81 years	707 (39.4%)	436 (40.6%)	271 (37.7%)	
≥ 81 years	249 (13.9%)	137 (12.7%)	112 (15.6%)	
Sex				0.617
Female	772 (43.1%)	468 (43.5%)	304 (42.3%)	
Male	1021 (56.9%)	607 (56.5%)	414 (57.7%)	
Race				0.992
American Indian/Alaska Native	13 (0.7%)	7 (0.7%)	6 (0.8%)	
Asian or Pacific Islander	103 (5.7%)	61 (5.7%)	42 (5.8%)	
Black	161 (9.0%)	99 (9.2%)	62 (8.6%)	
White	1516 (84.6%)	908 (84.4%)	608 (84.6%)	
Primary site				0.276
Head, face, neck	131 (7.3%)	70 (6.5%)	61 (8.5%)	
Up limb, shoulder	385 (21.5%)	225 (20.9%)	160 (22.3%)	
Lower limb, hip	1128 (62.9%)	693 (64.5%)	435 (60.6%)	
Trunk, NOS	149 (8.3%)	87 (8.1%)	62 (8.6%)	
Summary stage				0.606
Distant	178 (9.9%)	111 (10.3%)	67 (9.3%)	
Localized	1321 (73.7%)	783 (72.8%)	538 (74.9%)	
Regional	294 (16.4%)	181 (16.8%)	113 (15.7%)	
Grade				0.100
Moderately differentiated; Grade II	510 (28.4%)	298 (27.7%)	212 (29.5%)	
Poorly differentiated; Grade III	437 (24.4%)	246 (22.9%)	191 (26.6%)	
Undifferentiated; anaplastic; Grade IV	577 (32.2%)	367 (34.1%)	210 (29.2%)	
Well differentiated; Grade I	269 (15.0%)	164 (15.3%)	105 (14.6%)	
Laterality				0.617
Left-origin of primary	800 (44.6%)	475 (44.2%)	325 (45.3%)	
Not a paired site	206 (11.5%)	119 (11.1%)	87 (12.1%)	
Right-origin of primary	787 (43.9%)	481 (44.7%)	306 (42.6%)	
Chemotherapy				0.086
No/Unknown	1503 (83.8%)	888 (82.6%)	615 (85.7%)	
Yes	290 (16.2%)	187 (17.4%)	103 (14.3%)	
Radiation				0.850
Beam radiation	789 (44.0%)	475 (44.2%)	314 (43.7%)	
None/Unknown	1004 (56.0%)	600 (55.8%)	404 (56.3%)	
Directed surgery				0.555
Not performed	127 (7.1%)	73 (6.8%)	54 (7.5%)	
Surgery performed	1666 (92.9%)	1002 (93.2%)	664 (92.5%)	

Regarding the anatomical distribution of subcutaneous LMS, the most prevalent sites were the limbs, which encompassed both the upper and lower extremities (48.0%). This was followed by the trunk region (38.5%) and the head, face, and neck region (13.6%).

Treatment-wise, only 290 patients (16.2%) received chemotherapy, while 789 patients (44%) had a history of radiotherapy. Surgery was performed on 1666 patients (92.9%) as part of their management.

To facilitate analysis and validation, all patients were randomly divided into two groups: a training group consisting of 1075 cases and a validation group consisting of 718 cases.

### Independent predictors for patients with extremity soft tissue LMS

**Figure 2:**
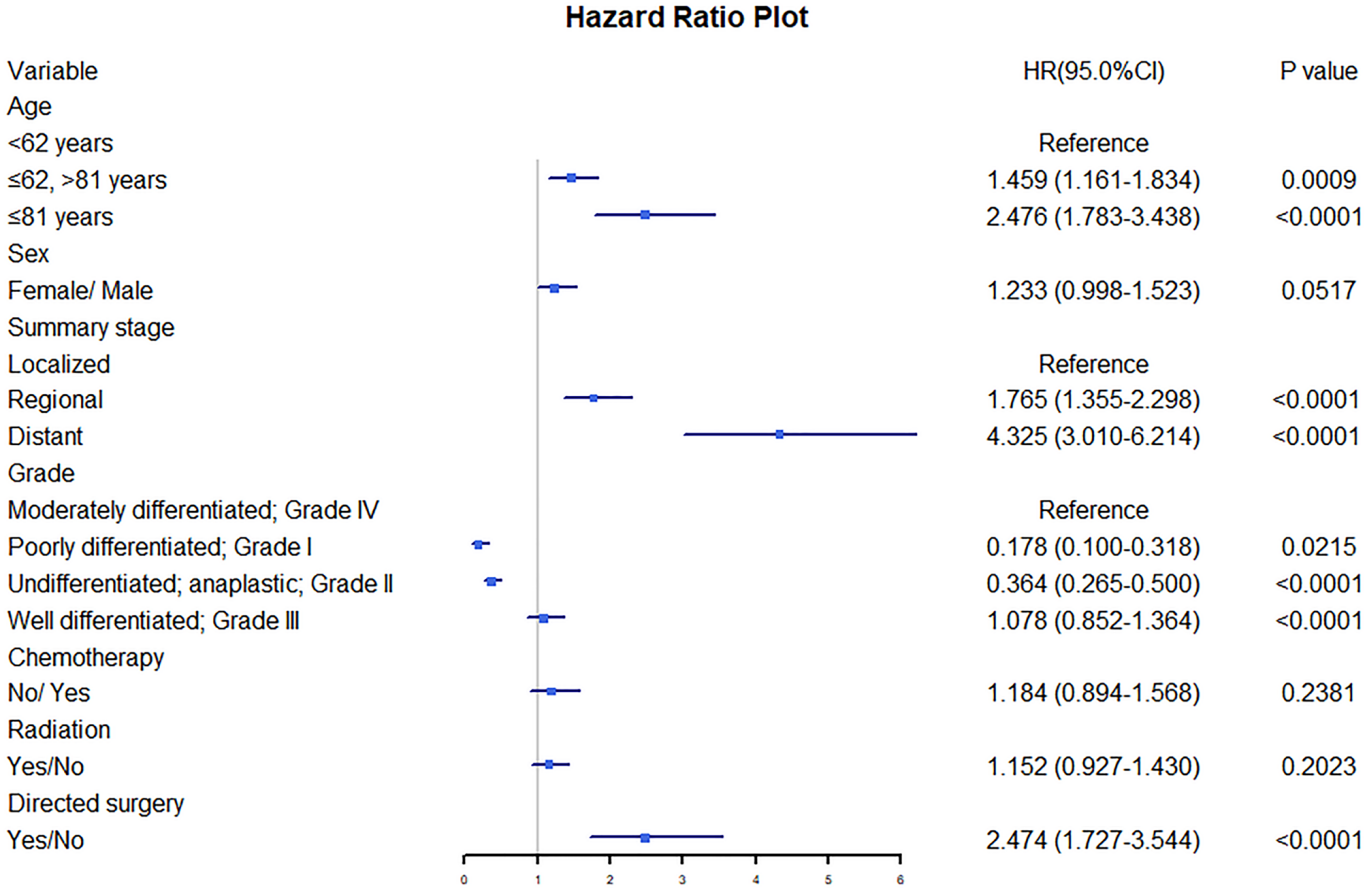
Cox multivariate analysis for CSS for patients with subcutaneous soft tissue LMS. HR: hazard ratio, CI: confidence interval.

The univariate analysis conducted in this study revealed that several factors were significantly associated with CSS in patients with subcutaneous LMS (Table 2). These factors included age, sex, summary stage, grade, chemotherapy, radiation therapy, and surgery, with *p* < 0.05. To further examine the CSS prognosis, a survival curve was constructed using the Kaplan–Meier method (Supplementary 1). Additionally, Cox multivariate analysis was employed to identify independent prognostic factors for patients with subcutaneous LMS (Fig. 2). The results indicated that age, summary stage, grade, and surgery emerged as independent prognostic factors associated with decreased CSS.

**Table 2:** Demographic and clinical characteristics of patients with subcutaneous LMS for the training cohort.

	Alive (N = 717)	Dead (N = 358)	*p*-value
Age			
< 62 years	363 (50.6%)	139 (38.8%)	< 0.001
≤ 62, > 81 years	274 (38.2%)	162 (45.3%)	
≥ 81 years	80 (11.2%)	57 (15.9%)	
Sex			
Female	288 (40.2%)	180 (50.3%)	0.002
Male	429 (59.8%)	178 (49.7%)	
Race			
American Indian/Alaska Native	3 (0.4%)	4 (1.1%)	0.431
Asian or Pacific Islander	42 (5.9%)	19 (5.3%)	
Black	62 (8.6%)	37 (10.3%)	
White	610 (85.1%)	298 (83.2%)	
Primary site			
Head, face, neck	49 (6.8%)	21 (5.9%)	0.121
Up limb, shoulder	163 (22.7%)	62 (17.3%)	
Lower limb, hip	445 (62.1%)	248 (69.3%)	
Trunk, NOS	60 (8.4%)	27 (7.5%)	
Summary stage			
Distant	21 (2.9%)	90 (25.1%)	< 0.001
Localized	598 (83.4%)	185 (51.7%)	
Regional	98 (13.7%)	83 (23.2%)	
Grade			
Moderately differentiated;Grade II	245 (34.2%)	53 (14.8%)	< 0.001
Poorly differentiated; Grade III	126 (17.6%)	120 (33.5%)	
Undifferentiated; anaplastic; Grade IV	195 (27.2%)	172 (48.0%)	
Well differentiated; Grade I	151 (21.1%)	13 (3.6%)	
Laterality			
Left- origin of primary	320 (44.6%)	155 (43.3%)	0.884
Not a paired site	80 (11.2%)	39 (10.9%)	
Right-origin of primary	317 (44.2%)	164 (45.8%)	
Chemotherapy			
No/Unknown	644 (89.8%)	244 (68.2%)	< 0.001
Yes	73 (10.2%)	114 (31.8%)	
Radiation			
Beam radiation	276 (38.5%)	199 (55.6%)	< 0.001
None/Unknown	441 (61.5%)	159 (44.4%)	
Directed surgery			
Not performed	16 (2.2%)	57 (15.9%)	< 0.001
Surgery performed	701 (97.8%)	301 (84.1%)

Notably, grade, which reflects the degree of pathological differentiation, displayed a significant correlation with the prognosis of subcutaneous LMS patients. Patients with distant metastases exhibited a substantially higher independent risk predictor for decreased CSS (HR 4.325, 95% CI 3.010–6.214, *p* < 0.001). Other significant independent risk predictors included age ≥ 82 years (HR 2.476, 95% CI 1.783–3.438, *p* < 0.001) and the absence of surgery (HR 2.474, 95% CI 1.727–3.544, *p* < 0.001).

### Construction of the nomogram and predictive model verification

**Figure 3:**
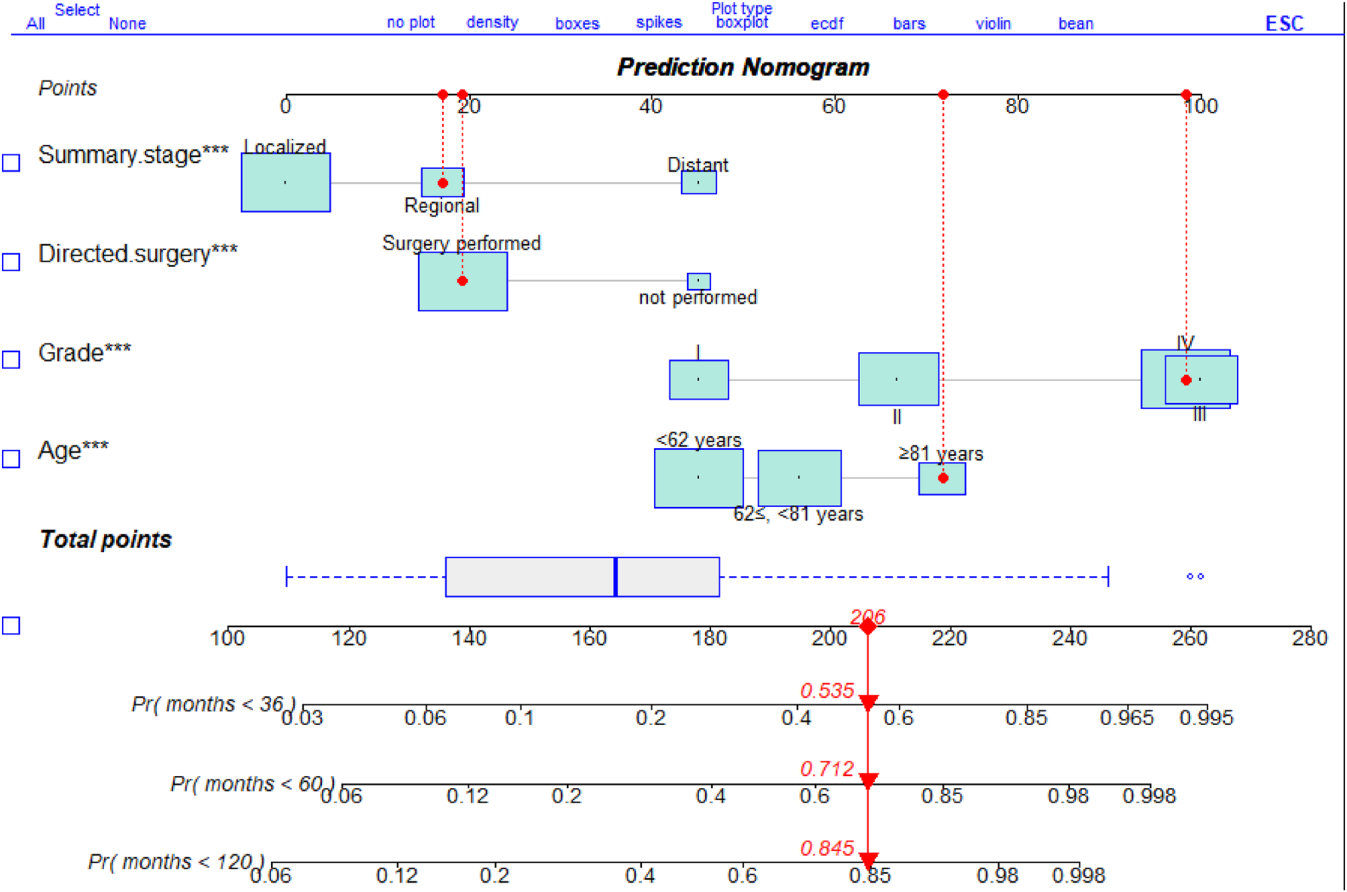
Nomogram predicting the probabilities of 3-, 5-, and 10-years cancer-specific death in the training cohort. Points are assigned for all 4 prognostic factors. For example, for an ≥ 81-years-old patient with Regional (Summary stage) and Grade III from this study that did undergo surgery, the total score is 206, and the corresponding probability of cancer specific disease-associated death at 3-, 5-, and 10-years are 0.535, 0.712 and 0.845.

Based on the identified prognostic factors, this study developed a nomogram model to predict the clinical outcome of patients with subcutaneous leiomyosarcoma (LMS) in the training cohort (Fig. 3). The performance of the model was evaluated using the concordance index (C-index), which yielded a value of 0.802 [95% CI 0.781–0.823], indicating a favorable predictive ability for CSS.

**Figure 4:**
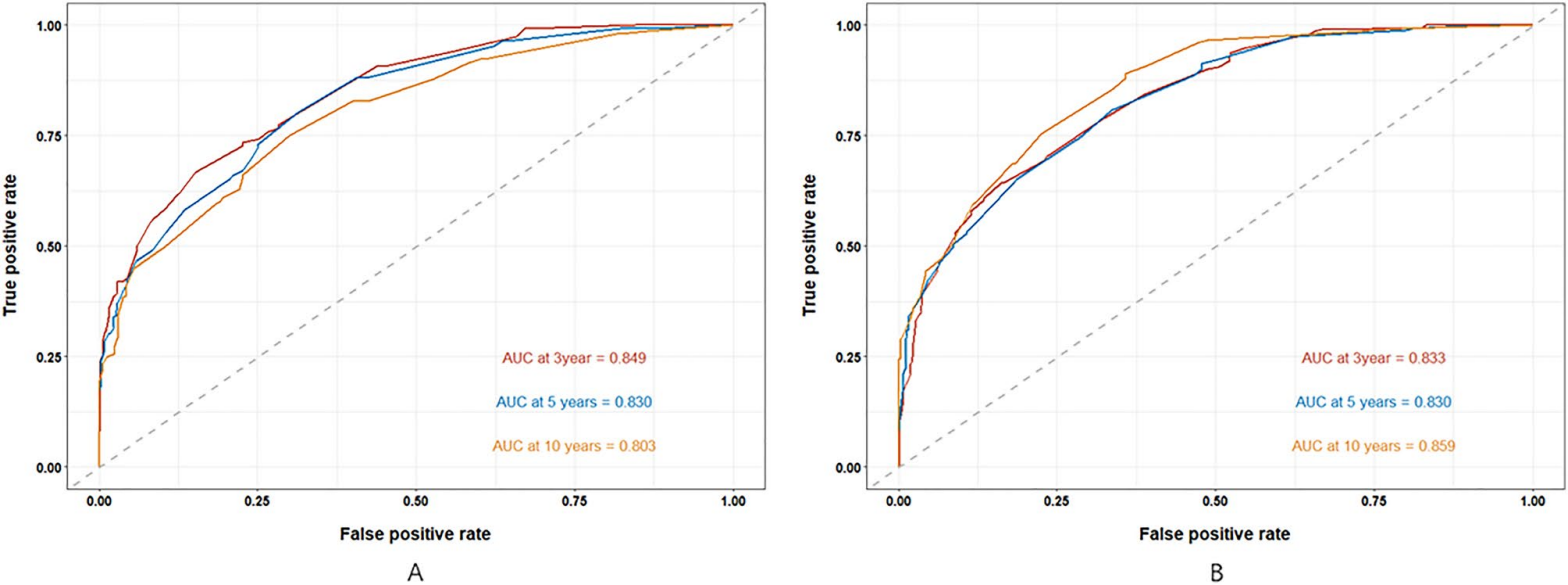
The 3-, 5-, and 10-years receiver operating characteristic curves patients with subcutaneous LMS in the training cohort (A) and validation cohort.

To further assess the discriminatory power of the nomogram, a receiver operating characteristic (ROC) curve was constructed. In the training cohort, the area under the curve (AUC) values for CSS prediction at 3, 5, and 10 years were 0.833, 0.830, and 0.859, respectively. Similarly, in the validation cohort, the C-index was 0.798[95% CI 0.768–0.829] and the corresponding AUC values for CSS prediction were 0.849, 0.830, and 0.803, respectively. These results indicate that the nomogram model exhibited superior predictive accuracy, highlighting its efficacy in prognosticating CSS (Fig. 4).

**Figure 5:**
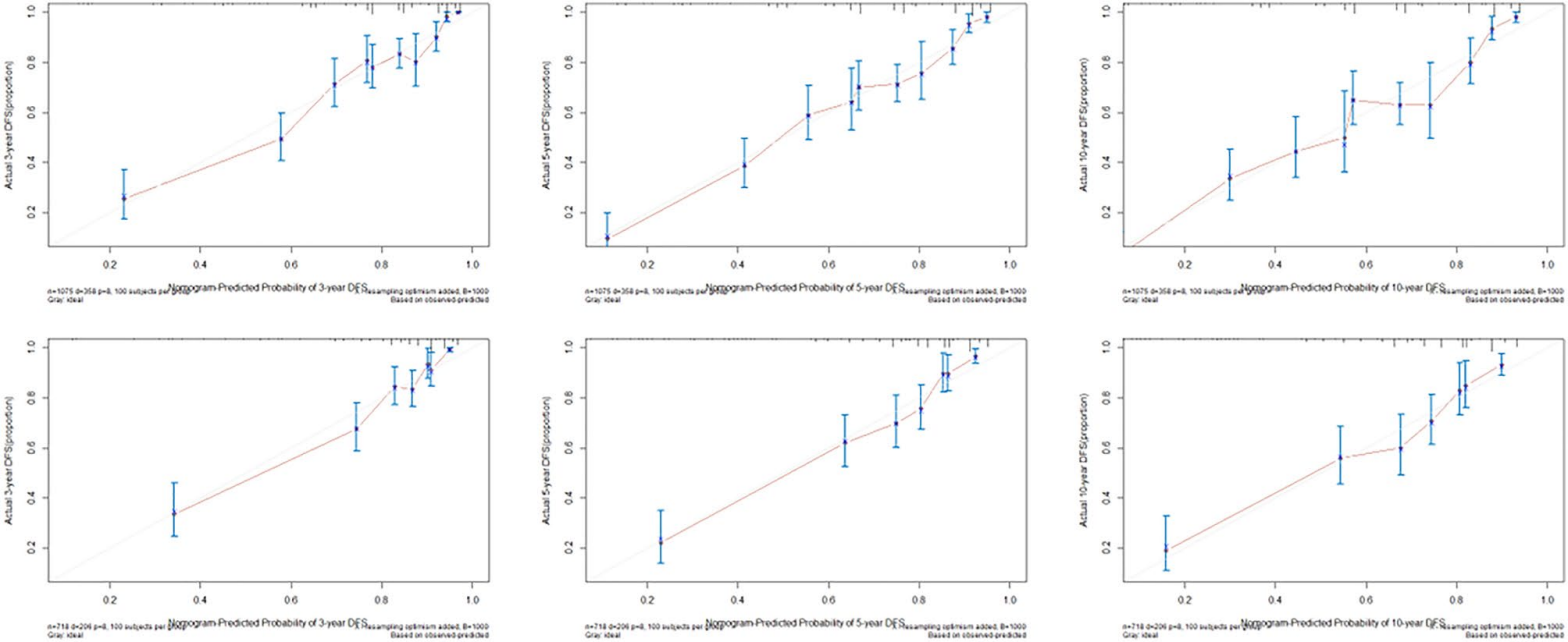
(**A**–**C**) Calibration curves showing the 3-, 5-, and 10-year CSS probabilities between nomogram predictions and actual probabilities in the training cohort in patients with subcutaneous LMS**.** (**D**–**F**) Calibration curves showing 3-, 5-, and 10-year CSS probabilities between nomogram predictions and actual probabilities in the validation cohort.

Furthermore, calibration plots were generated to assess the agreement between predicted and observed probabilities of 3-, 5-, and 10-year CSS in both the training and validation cohorts (Fig. 5). The plots demonstrate a high level of consistency between the predicted and observed probabilities, further confirming the reliable performance of the nomogram in predicting CSS.

### Decision tree model from prognostic nomogram

**Figure 6:**
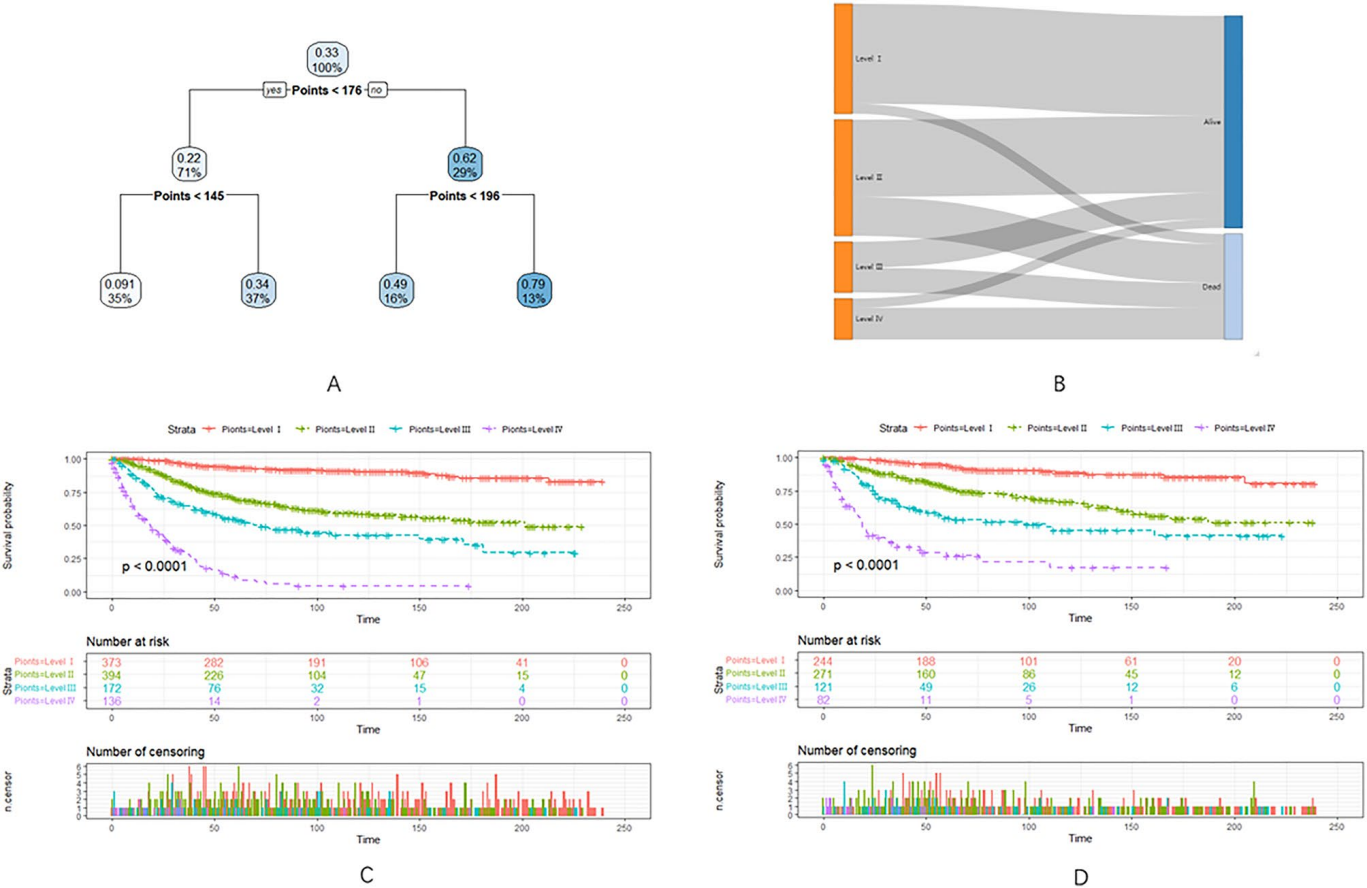
(**A**–**C**) Decision tree analysis model for overall survival of patients with patients with stage III malignant melanoma with elevated LDH. Decision tree (**A**); Decision tree analysis results into different risk groups (**B**); Survival curves for different risk groups for the training cohort (**C**); Survival curves for different risk groups for the valid cohort (**D**). Level I-IV: the low-risk group, the intermediate-low-risk group, the intermediate-high-risk group, and the high-risk group.

In the study, RPA categorized patients into four distinct risk subgroups based on the overall score obtained from the nomogram. These subgroups were classified as follows: the low-risk group with a total score less than 145, the intermediate-low-risk group with a score between 145 and 176, the intermediate-high-risk group with a score between 176 and 196, and the high-risk group with a score equal to or greater than 196. Notably, the probability of mortality varied significantly across the four risk subgroups, with respective rates of 9.1%, 34%, 49%, and 79%. These differences in mortality rates were found to be statistically significant in both the training cohort and the validation cohort, with all *p* < 0.01, as depicted in Fig. 6.

## Discussion

In this study, we conducted a comprehensive analysis of 1,793 patients diagnosed with subcutaneous LMS using data from the SEER database spanning from 2000 to 2019. Our study deviates from previously published literature, as we discovered discrepancies in the applicability of the AJCC 7th edition staging system for soft tissue sarcoma in current clinical practice. Consequently, we revised the staging system by incorporating the latest edition. After excluded the ICD-O-3 codes C49.3 (thorax), C49.4 (abdomen), and C49.5 (pelvis) to accurately classify soft tissue sarcomas occurring in the abdominal, thoracic, and pelvic visceral organs, we proceeded to analyze the clinical characteristics and treatment information of the remaining subcutaneous LMS cases. Through this analysis, we identified the key risk factors affecting CSS in subcutaneous LMS. Additionally, we calculated the percentage of 3-, 5-, and 10-year survival rates to provide a comprehensive understanding of long-term outcomes. Of particular importance, we employed four variables—age, summary stage, grade, and surgery—to develop a prognostic nomogram. This nomogram serves as a visual tool to estimate individual patient outcomes based on their specific characteristics. Furthermore, to enhance the clinical applicability of our findings, we constructed a decision tree model utilizing these four variables. This model provides a practical and user-friendly framework to aid clinicians and patients in making informed treatment decisions. In our study, we aim to contribute valuable insights into risk stratification and prognostic assessment for subcutaneous LMS, ultimately improving the quality of care and personalized treatment approaches for affected individuals.

In this study, we observe a heightened mortality risk among elderly patients with subcutaneous leiomyosarcoma (LMS), as substantiated by existing literature^11,15,16^. Older individuals, often marked by unique lifestyles encompassing dietary and exercise habits, are also prone to additional underlying health issues and suboptimal conditions. These age-related factors possess the potential to significantly impact disease progression and survival rates. Furthermore, the treatment preferences of both physicians and patients may be influenced by these factors, subsequently contributing to fluctuations in the specific mortality rate associated with subcutaneous LMS. Previous research has indicated that the specific primary locations of soft tissue sarcomas can lead to varying treatment outcomes and prognosis^17–20^. However, in the univariate analysis, we did not observe a statistically significant association between different sites of subcutaneous LMS and CSS.

Numerous studies have highlighted the significance of tumor grades as independent prognostic factors in soft tissue sarcoma^21–23^. One study specifically emphasized that high-grade subcutaneous leiomyosarcoma (LMS) is primarily associated with local recurrence and negatively impacts the median cancer-specific survival (CSS) of LMS patients^24^. In our study, we also observed that a high histological grade was a significant predictor of poor prognosis in subcutaneous LMS. While the risk of lymph node metastasis is relatively low, leiomyosarcoma still poses a potential for distant metastasis^25–27^. Compared to dermal LMS, subcutaneous LMS demonstrates a higher likelihood of local recurrence or metastasis, leading to lower overall survival rates. Additionally, subcutaneous LMS more frequently invades the lungs, breasts, and pleura^6,7,28,29^. Our current study further supports these findings by revealing a significantly lower CSS in patients with any distant metastasis, indicating an unfavorable prognosis for individuals with subcutaneous LMS.

Surgical resection is widely considered the optimal treatment for LMS, as it not only prolongs patients' survival but also enhances their quality of life^25,30,31^. However, consensus is lacking concerning surgical approaches for subcutaneous leiomyosarcoma (LMS). Traditional guidelines advocate for radical surgery with a 3–5 cm margin; nevertheless, recent studies suggest that reducing the surgical margin to 1 cm does not increase the local recurrence rate^32^. Although the SEER database lacks detailed explanations of surgical specifics, the results still reveal that individuals with subcutaneous LMS who underwent surgery experienced prolonged CSS. While radiation therapy can provide pain relief and achieve effective local control of sarcoma, it was not found to be independently associated with CSS in our study participants. This aligns with previous research indicating that radiotherapy does not significantly impact the likelihood of local recurrence in LMS patients^23^. Chemotherapy may be considered for cases of metastatic LMS when complete surgical removal is not feasible. While retrospective studies indicate that subcutaneous LMS patients receiving chemotherapy have demonstrated therapeutic effects, our study did not show its decisive role in treatments^33–35^. Nevertheless, radiation and chemotherapy exhibit some effects, serving as significant adjuvant measures to reduce subcutaneous LMS recurrence. Further research is needed to determine the optimal treatment approach.

Based on the above prognostic factors, a novel nomogram model was developed to predict cancer-specific survival (CSS) in patients with subcutaneous LMS. This study demonstrated that the nomogram model exhibited superior predictive ability and accuracy in both the training and validation cohorts, with C-indexes of 0.802 and 0.798, respectively. Emphasizing patient involvement in decision-making, the nomogram underscores the need for thorough communication between physicians and patients to ensure a comprehensive understanding of the potential benefits and risks associated with surgery, facilitating a mutually agreed-upon decision. Rather than being an independent decision-making basis, the model should function as a decision-support tool. In its application, physicians ought to consider individual patient variations, combining professional judgment with predictions from the flowchart to formulate the most suitable treatment plan.

To further classify patients based on their risk levels, a decision tree model was employed using the nomogram in the training cohort. The risk subgroups identified were the low-risk group (< 145), intermediate-low risk group (≥ 145 and < 176), intermediate-high risk group (≥ 176 and < 196), and high-risk group (≥ 196). As the total score increased within each risk subgroup, the probability of death gradually escalated. Notably, significant differences were observed among the four risk subgroups in both the training and validation cohorts. This observation indicates that the efficacy of integrating the decision tree model with the nomogram, presenting a promising and practical strategy for the assessment of CSS in patients afflicted with subcutaneous LMS. By combining the strengths of both models, a comprehensive and nuanced evaluation can be achieved, offering clinicians a valuable tool to navigate the complexities of predicting cancer-specific outcomes. This integrated approach not only enhances the predictive accuracy but also provides a more holistic understanding of the factors influencing cancer-specific survival in subcutaneous LMS cases. The synergy between the decision tree model and the nomogram contributes to a robust and versatile framework, facilitating a nuanced interpretation of patient-specific prognostic information and enabling more informed and personalized treatment decisions.

There are certain limitations to this study that must be addressed. First, the study was designed using data from SEER cases, which did not fully account for all potential risk factors; second, because this was a retrospective study, there may have been some selection biases and errors in record entry; thus, prospective studies with a large sample size from multiple institutions are required; and third, an external validation cohort is required to validate the model.

### Supplementary Information

Supplementary Information.

## Data Availability

The datasets used and analyzed during the current study are available from the SEER*stat software (https://seer.cancer.gov/resources/).
