# Supplementary material for: A novel nomogram and recursive partitioning analysis for predicting cancer-specific survival of patients with subcutaneous leiomyosarcoma
Source: Sci Rep. 2024 Feb 4;14:2861. doi: 10.1038/s41598-024-53288-6 (PMC10838934; doi:10.1038/s41598-024-53288-6)
Supplement: Supplementary file 1 — Supplementary Information. [file 41598_2024_53288_MOESM1_ESM.docx]

**Supplemental Figure**

| **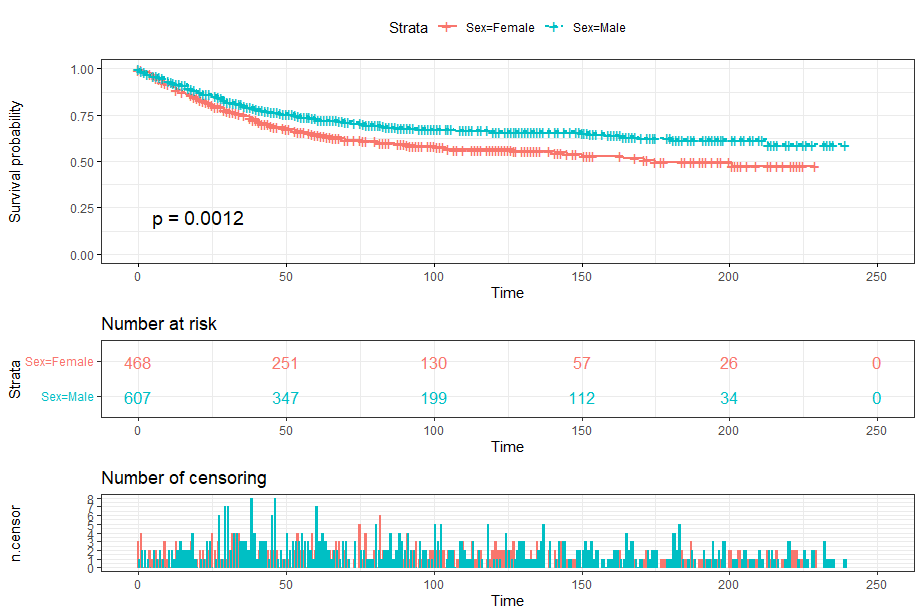**  **A** | **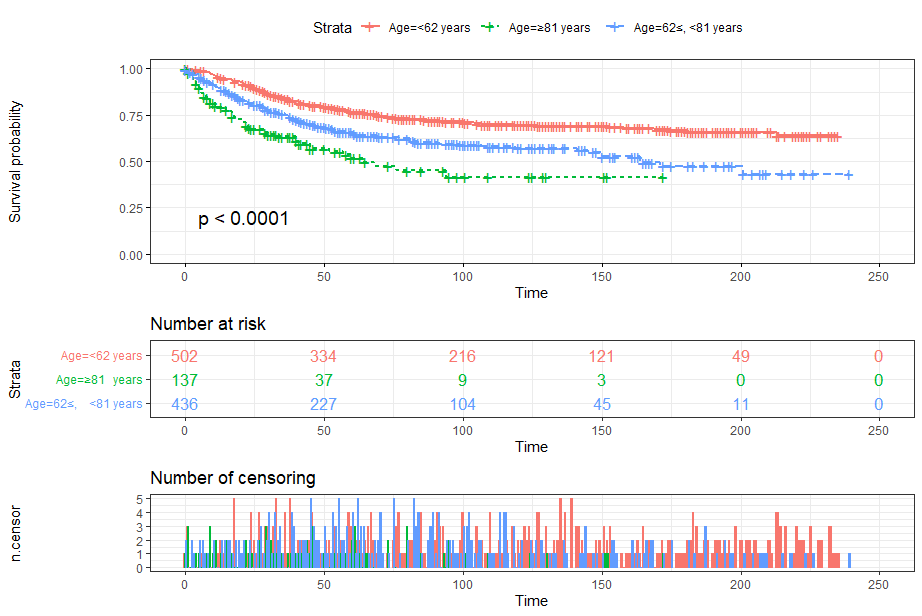**  **B** |
| --- | --- |
| **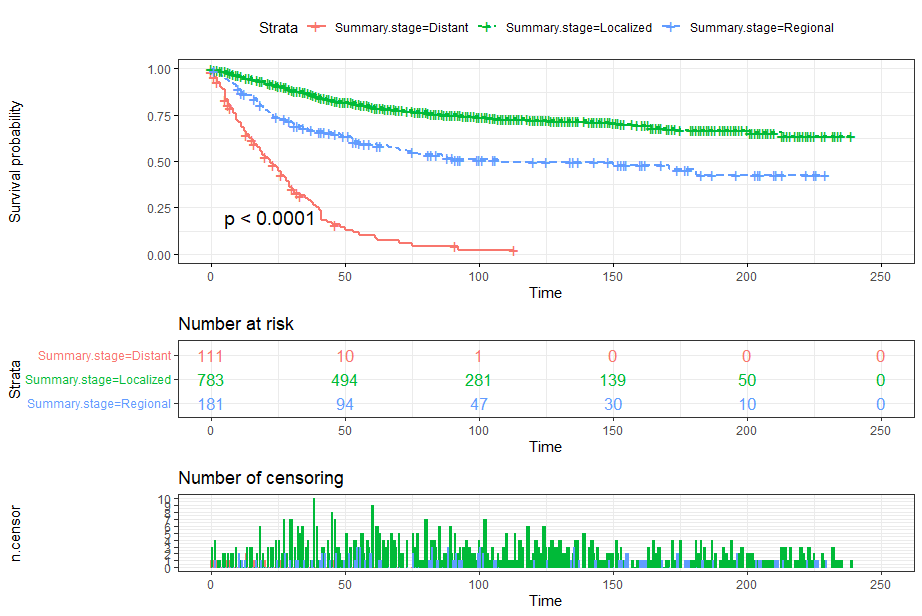**  **C** | **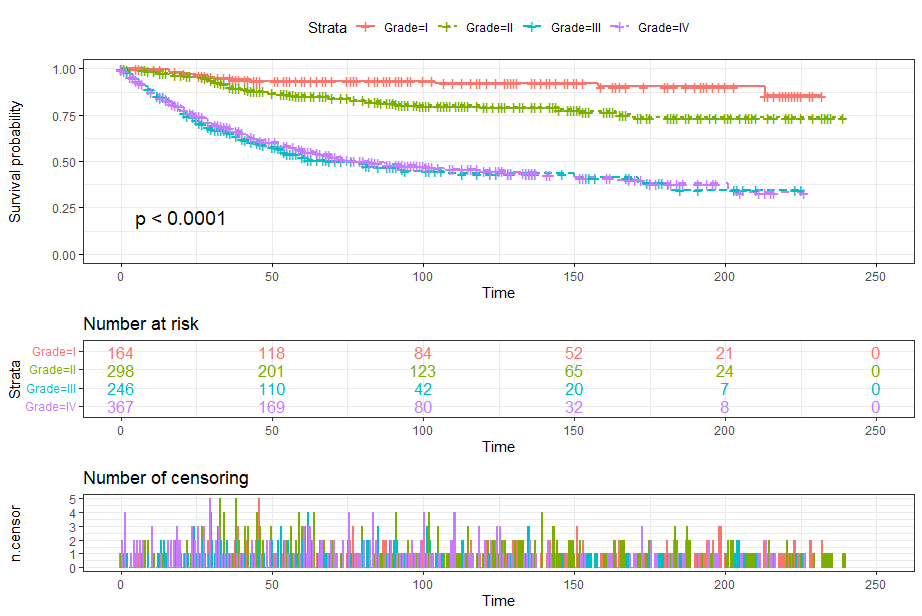**  **D** |
| **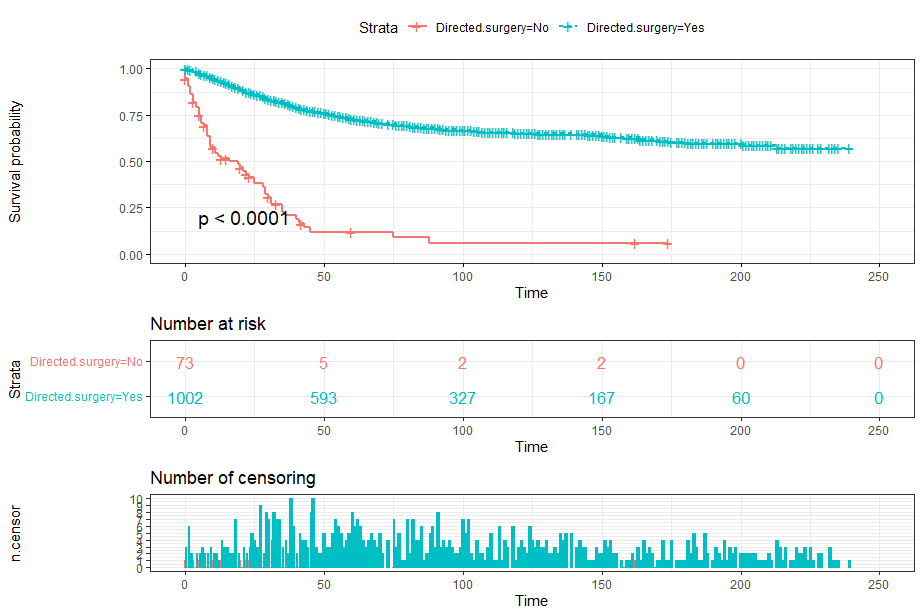**  **E** | **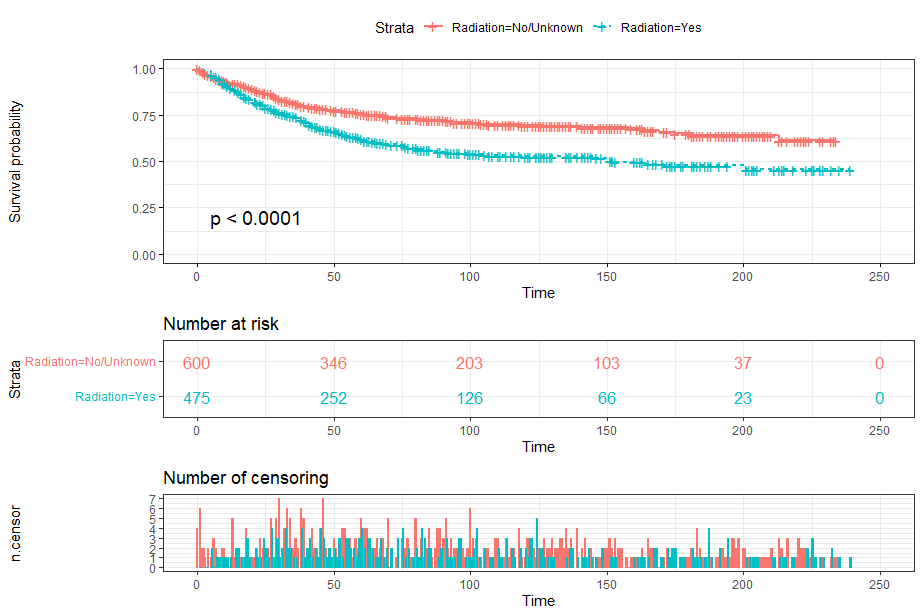**  **F** |
| **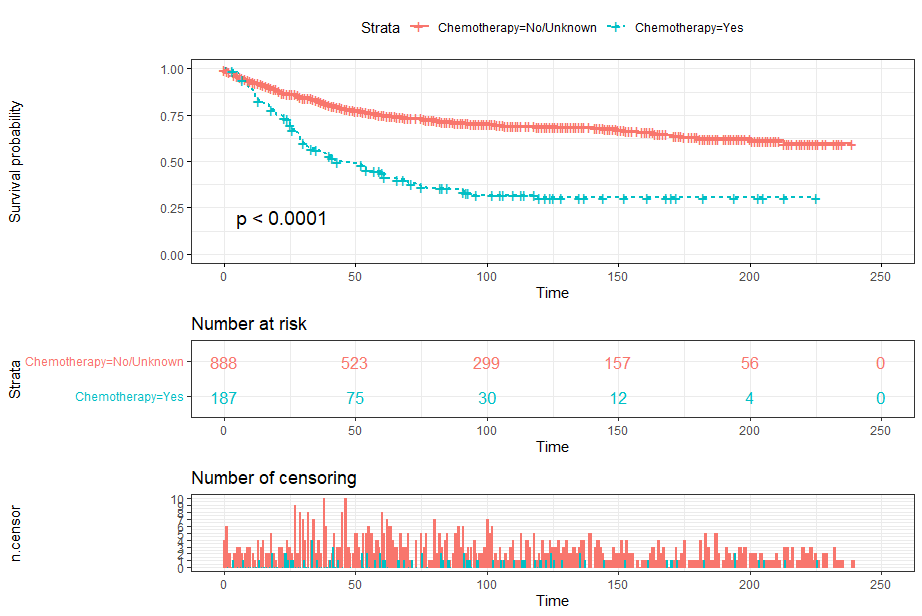**  **G** |  |

Supplementary Fig: Kaplan-Meier method estimated CSS in patients with **subcutaneous LMS.** stratified by Sex (A); Age (B); Summary stage (C); Grade (D); Directed surgery (E); Radiation (F); Chemotherapy (G).
